# Nano-Second Laser Interference Photoembossed Microstructures for Enhanced Cell Alignment

**DOI:** 10.3390/polym13172958

**Published:** 2021-08-31

**Authors:** Alba Martínez, Sandra González-Lana, Laura Asín, Jesús M. de la Fuente, Cees W. M. Bastiaansen, Dirk J. Broer, Carlos Sánchez-Somolinos

**Affiliations:** 1 Instituto de Nanociencia y Materiales de Aragón (INMA), CSIC-Universidad de Zaragoza, Advanced Manufacturing Laboratory, Departamento de Física de la Materia Condensada, C./Pedro Cerbuna 12, 50009 Zaragoza, Spain; albamartinez2292@gmail.com (A.M.); sangolan@unizar.es (S.G.-L.); 2 BEONCHIP S.L., CEMINEM, Campus Rio Ebro. C./Mariano Esquillor Gómez s/n, 50018 Zaragoza, Spain; 3 Instituto de Nanociencia y Materiales de Aragón (INMA), CSIC-Universidad de Zaragoza, C./Pedro Cerbuna 12, 50009 Zaragoza, Spain; lasin@unizar.es (L.A.); jmfuente@unizar.es (J.M.d.l.F.); 4 CIBER in Bioengineering, Biomaterials and Nanomedicine (CIBER-BBN), Spain; 5 Faculty of Chemistry and Chemical Engineering, Eindhoven University, P.O. Box 513, 5600 Eindhoven, The Netherlands; C.W.M.Bastiaansen@TUE.NL (C.W.M.B.); D.Broer@tue.nl (D.J.B.)

**Keywords:** photopolymers, photoembossing, interference holography, microstructuring, cell alignment, cell morphology

## Abstract

Photoembossing is a powerful photolithographic technique to prepare surface relief structures relying on polymerization-induced diffusion in a solventless development step. Conveniently, surface patterns are formed by two or more interfering laser beams without the need for a lithographic mask. The use of nanosecond pulsed light-based interference lithography strengthens the pattern resolution through the absence of vibrational line pattern distortions. Typically, a conventional photoembossing protocol consists of an exposure step at room temperature that is followed by a thermal development step at high temperature. In this work, we explore the possibility to perform the pulsed holographic exposure directly at the development temperature. The surface relief structures generated using this modified photoembossing protocol are compared with those generated using the conventional one. Importantly, the enhancement of surface relief height has been observed by exposing the samples directly at the development temperature, reaching approximately double relief heights when compared to samples obtained using the conventional protocol. Advantageously, the light dose needed to reach the optimum height and the amount of photoinitiator can be substantially reduced in this modified protocol, demonstrating it to be a more efficient process for surface relief generation in photopolymers. Kidney epithelial cell alignment studies on substrates with relief-height optimized structures generated using the two described protocols demonstrate improved cell alignment in samples generated with exposure directly at the development temperature, highlighting the relevance of the height enhancement reached by this method. Although cell alignment is well-known to be enhanced by increasing the relief height of the polymeric grating, our work demonstrates nano-second laser interference photoembossing as a powerful tool to easily prepare polymeric gratings with tunable topography in the range of interest for fundamental cell alignment studies.

## 1. Introduction

Surface polymeric microstructures are key elements for the development of different scientific and technological areas [1]. Polymeric microstructures are used, for example, as intermediate elements to create the building blocks and connections of microprocessors [2]. They are also used to create microlenses, waveguides, or diffractive elements to enhance the performance of photonic devices such as solar cells, liquid crystal or light-emitting diode displays [1,3,4]. In the area of biomedicine, it is well recognized that surface topographic microstructures play a major role in regulating important aspects of cell behavior (migration, cell adhesion, proliferation, differentiation) and morphology, which are key to achieve functional tissue constructs for tissue regeneration and transplants, as well as the successful preparation of artificial in vitro biomimetic biological systems [5,6,7,8,9,10,11].

Given their relevance, a large effort has been done over the last decades to advance towards surface relief polymeric structures with a wide variety of patterns, increasing resolution and complexity, and produced in a wide range of materials. Microstructuring technologies based on light have largely contributed to this progress mainly due to the concurrent development of advanced photonic systems and photosensitive materials, as well as our increased knowledge of the interaction between light and matter [4]. In the most traditional photolithographic approach, a mask with transparent and opaque regions is used to selectively irradiate certain regions of a photopolymer layer, introducing a solubility change in the exposed areas. The use of an appropriate solvent selectively removes certain areas of the film and generates a surface relief structure with the same features as the employed mask [3,12,13,14].

Besides conventional mask photolithography making use of wet etching steps, other solvent-free methodologies have been developed. Among them, photoembossing relies on polymerization-induced diffusion to generate surface relief structures without the need of any solvent developing step. Photoembossing typically makes use of photoresists essentially comprising a polymeric binder, multireactive monomers and a photoinitiator. The photoresists usually employed are glassy at room temperature (RT), leading to tack-free films that allow photolithographic mask contact in the exposure step carried out at this temperature [15]. The illumination of thin films of these photoresists through the mask generates reactive species in the exposed areas. The non-reactive polymeric binder provides mechanical stability to the photosensitive film while the low-molecular-weight monomers could diffuse and react; however, with light exposure carried out at a temperature at RT, below the glass transition temperature (T_g_), polymerization and diffusion are largely inhibited, leading to a latent image of reactive species. The heating of the sample above the T_g_ of the system provides mobility to the molecular species, and therefore diffusion and polymerization is significantly enabled. The net flux of materials from the non-illuminated regions to the irradiated ones leads to the development of surface relief structures with topographical features that are mainly controlled by the diffusion parameters of the reactive, as well as free, surface energy [16,17,18,19,20,21,22,23,24,25,26].

Besides the fabrication of diffraction gratings for light management applications, photoembossing has been demonstrated to have potential in the fabrication of microlense arrays whose optical characteristics (e.g., dimensions and focal length) can be easily controlled by the appropriate selection of processing parameters [27]. Photoembossing has also been explored for the preparation of fibers showing optical effects [28], liquid-filled microcavities with potential use in the large-area production of electrophoretic displays [29] and biocompatible surfaces for controlled cell growth [30,31].

Besides using a photolithographic mask, the photoembossing of surface relief structures have also been demonstrated using interference lithography (IL), without the need for any mask in contact with the sample. The interference of two coherent light beams of actinic light leads to a periodic light pattern in the region of the sample that enables the formation of a latent image of reactive species. Control of the angle and the phase difference between the beams, as well as polarization and amplitude, enables the generation of complex well-defined periodic patterns. The use of interference patterns, either using continuous wave or nanosecond pulsed lasers, has been demonstrated to lead to similar results, in terms of height, to those obtained by contact-mask exposure to actinic light of similar wavelength [32]. Advantageously, when using ns pulse lasers, given the short time exposure, in comparison to CW recording, the system is largely insensitive to vibrations and slow movements, opening the possibility of integrating this technique in roll-to-roll processes to manufacture surface microstructures.

IL offers an additional, not yet exploited, advantage. While the creation of structures by means of mask-assisted photoembossing requires the exposure step to be carried out at temperatures below the T_g_ to allow contact of the mask and avoid its adhesion to the sample; in IL assisted photoembossing, being a non-contact technique, exposure of the sample directly at the development temperature could be carried out. In this paper, we explore this possibility and perform a systematic study of a modified photoembossing protocol consisting of a pulsed holographic exposure carried out directly with the sample at the surface development temperature. The surface relief structures, so generated, are compared with those obtained by using the conventional photoembossing protocol based on exposure at room temperature (RT) and a subsequent heating step for the development of the structure. The influence of the development temperature, the effect of the photoinitiator concentration and the period of the illumination pattern on the height of the microstructures using this modified photoembossing protocol has been investigated. Finally, the potential of the newly generated structures as cell-aligning substrates is explored.

## 2. Materials and Methods

### 2.1. Materials

The polymer binder polybenzylmethacrylate (PBMA) was purchased from Scientific Polymer Products Inc. (Ontario, NY, USA) (weight-average molecular weight-Mw-70,000 g mol^−1^) and the multireactive monomer dipentaerythritol penta-/hexa-acrylate (DPPHA) from Aldrich (Saint Louis, MO, USA). The photoinitiator Irgacure 369 (IRG369) was purchased from Aldrich (Saint Louis, MO, USA). All the materials were used as received without further purification.

### 2.2. Photopolymer and Film Formation

The photopolymer was prepared by dissolving the polymeric binder PBMA and the monomer DPPHA in a 1:1 weight ratio in propylene glycol methyl ether acetate (PGMEA) that acts as a solvent. PGMEA was added into the mixture to reach a 1:1 weight ratio between PGMEA and the sum of the weights of PBMA and DPPHA of the mixture. Above this, different percentages of the photoinitiator were added and dissolved. The resultant solution was spin-coated on ozone-treated glass substrates. The so-prepared photosensitive films were annealed at 80 °C for 10 min to ensure complete solvent evaporation.

### 2.3. Interference Lithography (IL) Setup

IL exposure was carried out using the 355 nm linearly polarized light from a pulsed Nd:Yag laser (4 ns pulses) coupled to second and third harmonic modules. The beam of the laser, having a vertical polarization, was split into two equal intensity beams that were made to interfere at the position of the photosensitive film that was mounted on a hot stage with optical access. The angle between the two beams was adjusted to obtain an interference period of 9 μm or 4.5 μm, depending on the setup configuration selected.

### 2.4. Photoembossing Protocols

For the conventional photoembossing protocol, the exposure of the photopolymer film to the UV intensity interference pattern was performed at RT. Then the samples were immediately heated to high temperatures (60, 80 or 100 °C) for 10 min for relief development. For the modified photoembossing protocol, exposure was directly carried out at the selected temperature (again 60, 80 or 100 °C), and the samples were kept at this same temperature for 10 min for relief development. In both cases, conventional and modified photoembossing protocols, after the relief development step at high temperature, the samples were fast cooled down to RT and fully cured and fixed by performing a flood exposure step at RT using a UV Exfo Mercury lamp (Gentec, Nivelles, Belgium) (320–390 nm; 10 mW/cm^2^) for 10 min, followed by a heating step at 80 °C for 10 min.

### 2.5. Surface Topography Characterization

Topographic characterization of the surface relief structures was performed using a Dual Sensofar PLμ2300 microscope (Sensofar, Madrid, Spain) working in confocal mode.

### 2.6. Scanning Electron Microscopy (SEM) Characterization

SEM images were obtained using a QUANTA-FEG 250, FEI (Eindhoven, The Netherlands).

### 2.7. Optical Microscopy Characterization

Optical microscopy images were obtained using an inverted microscope: Nikon Eclipse TE2000-S provided with a digital camera head Slight DS-Fi1c.

### 2.8. Cell Culture and Cell Imaging

Kidney epithelial cells (Vero) were cultured in Dulbecco’s modified Eagle’s medium (DMEM, Lonza, BE12-614F) supplemented with 10% fetal bovine serum (FBS), 1% glutamine and 1% a mixture of penicillin and streptomycin. Cells were maintained under 37 °C and 5% CO_2_ conditions. Photoembossed patterned substrates were sterilized in a laminar flow cabinet by UV light exposure for 20 min each side.

Vero cells were split up from culture flasks with trypsin and counted in a Neubaüer chamber. A total of 500 μL of a 10^5^ cells/mL suspension in complete medium were seeded on each micropatterned substrate placed in 24 well-plate wells. After an incubation period of 6 h at 37 °C and 5% CO_2_ conditions, for the samples selected for optical microscopy, the medium was removed, and the cells were fixed with 4% paraformaldehyde (PFA) for 15 min at RT. After that, cells were stained for non-specific proteins using 0.5% Coomassie blue to enhance the contrast and better visualize cell morphology and elongation state.

Cell samples for SEM imaging were fixed adding to the culture medium 500 μL of 4% glutaraldehyde (GTA) in 0.2 M sodium cacodylate buffer (SCB) (pH = 7.2) for 2 h at 4 °C. The samples were then conserved in 2% GTA in 0.1M SCB (pH = 7.2) overnight at 4 °C. Then, the samples were washed with 0.1 M SCB (pH = 7.2) twice and dehydrated by immersing them serially in increasing MeOH concentration solutions (30% twice for 5 min, 50% twice for 5 min, 70% twice for 5 min and 100% twice for 10 min). The samples were finally immersed in 100% MeOH anhydrous twice for 5 min and conserved in it at 4 °C until SEM imaging acquisition. Samples were air-dried and observed using SEM.

### 2.9. Cell Alignment Measurements

The cell alignment distribution was calculated from the average angle, θ, between the major axis of the cell body and the direction of the lines of the grating microstructure. The transparency of the substrates allowed the direct measurement of the cell alignment angle using images obtained with optical microscopy. Images were taken at 10× magnification and the relative angle of the cell to the direction of the microstructure and aspect ratio were calculated using Fiji software [33]. Cell alignment angles were normalized to a single quadrant with resulting angles between a range of 0° and 90°. An unpaired Student t-test was performed to determine statistical significance. Each data set consists of measurements of 100 cells across two experiments.

## 3. Results and Discussion

**Figure 1:**
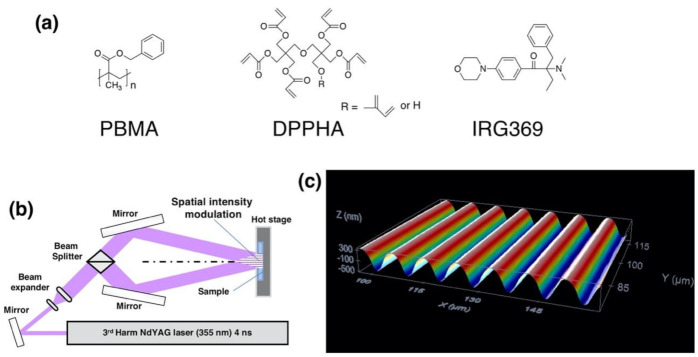
(**a**) Photopolymer components molecular structure. (**b**) Interference lithography setup. (**c**) Surface-relief topography of a 9 μm period grating generated using IL setup and a conventional photoembossing protocol comprising an exposure step at RT (energy dose: 3.6 mJ/cm^2^), followed by a heating step to high temperature (development temperature: 80 °C).

A photopolymer blend, of which the composition has been previously reported, is used to explore the impact of carrying out the pulsed holographic exposure step directly at the development temperature (modified photoembossing protocol) on the generation of surface relief structures [18,32]. The blend comprises PBMA as a polymeric binder and a multifunctional acrylate, DPPHA, in a 1:1 ratio, dissolved in PGMEA as described in the experimental section. Additionally, the blend contains, if not differently specified, 5 wt% of a radical photoinitiator, IRG369, to photosensitize the material (Figure 1a).

### 3.1. Conventional Photoembossing Protocol

We have first performed a reference study to characterize the surface relief structures obtained by using the conventional photoembossing protocol in which light exposure is first carried out at RT to later perform a thermal development step at elevated temperature. Following this procedure, 9 μm thick photopolymer films obtained by spin coating from the just described PGMEA-based solution were exposed at RT to the interference pattern of the IL setup shown in Figure 1b. The angle between the two beams was adjusted to lead to an interference period of 9 μm. We have exposed samples to different energies that are delivered to the sample as a single light pulse. As previously described, samples processed in this way and kept at this same RT develop no significant relief after exposure, even after several days [18,32]. The heating of the sample immediately after the exposure step to high temperature (e.g., 80 °C), above the T_g_ of the system, for 10 min leads to the development of periodic surface structures (Figure 1c as a representative example) showing the same period of the interference pattern. This thermal development step, done by placing the sample on a hot plate at the set temperature, is followed by a flood-exposure with UV actinic light at RT and subsequent heating, using a hot plate, at 80 °C for 10 min that leads to the full fixation of the sample. It was checked that this fixation step does not introduce significant changes in the structure. The height of the periodic grating, defined as the difference height between the maxima and minima, is the same when compared to the sample prior to the fixation step, within the experimental error. Besides, the structure is stable over periods of years, based on previous studies carried out in our laboratories.

**Figure 2:**
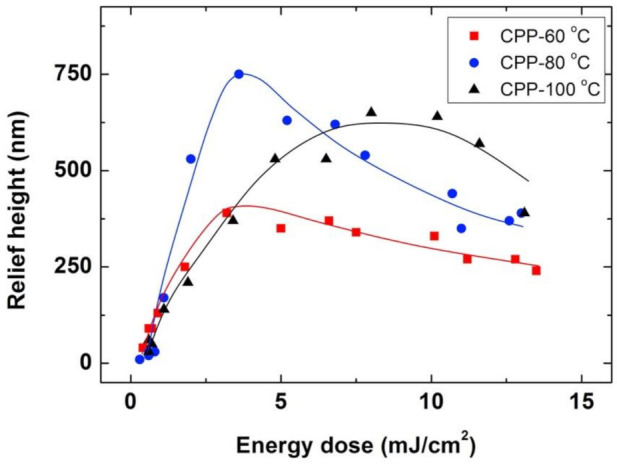
Conventional photoembossing protocol (CPP) comprising an exposure step at RT using IL (single pulse exposure and 9 μm period grating), followed by a heating step to high temperature. Relief height as a function of energy dose for different temperatures: 60 °C (■), 80 °C (⚫) and 100 °C (▲). Lines are drawn to guide the eye.

Figure 2 shows the height of the periodic structures for samples prepared using the conventional protocol. Different energy exposures and three different development temperatures, specifically, 60, 80 and 100 °C, have been employed. For the three different temperatures, we find that for small energy doses, the surface relief amplitude increases as the energy dose increases to reach an optimum height (390 nm for 60 °C, 750 nm for 80 °C and 650 nm for 100 °C). For doses higher than the optimum one, the amplitude of the surface relief decreases again with increasing energy. Despite this, qualitative behavior is similar for the three temperatures, the optimum height is different and reached for different conditions, with a highest structure of 750 nm reached for a dose of 3.6 mJ/cm^2^ and a development temperature of 80 °C.

The observed phenomenology can be understood by taking into account the diffusion of reactive species induced by light-induced polymerization as previously described by Broer and coworkers [15,16,17,18]. Essentially for this system, the non-reactive polymeric binder is taken as a stationary phase while the monomers can migrate within the film and become fixed in the exposed areas, therefore leading to more pronounced relief structures. In order to understand the differences between systems and conditions, aspects such as the polymerization-induced monomer concentration gradient, diffusivity differences, reactivities and interaction between components, as well as surface free energy, need to be considered [16,17].

Exposure to the pulsed spatially structured light locally generates, in our case, free radicals in the high-intensity regions of the interference pattern. These free radicals are fragments of the photo-dissociated photoinitiator and the first addition products to the monomers that could be reached taking the diffusion limits into consideration. These first addition products, taking the chain-addition polymerization principle into account, are also free radicals, intrinsically reactive, but again hindered by diffusion limitations as long as the sample is in its vitrified state below the glass transition temperature. Electronic paramagnetic resonance (EPR) studies revealed that, in the vitrified state, the free radicals are known to remain stable for days [34,35]. The heating of the sample promotes molecular mobility; monomers can reach the reactive sites by diffusion, and thus, polymerization can take place selectively in the high-intensity regions of the interference pattern. As a result, because of the monomer reaction, a compositional gradient is induced between the high- and low-intensity regions of the interference pattern that promotes the diffusion of monomers. Below the optimum dose, the amount of excited photoinitiator is low, and this results in the low incorporation of monomers to the polymeric network in the high-intensity regions and thus, low surface relief heights of the structure. Increasing the dose, results in higher conversion of monomers in the high intensity regions and thus, higher relief structures.

Light doses above the optimum lead to a high concentration of radicals in the high-intensity regions of the interference pattern that can produce a high, excessive degree of crosslinking in these regions that diminishes diffusivity of the monomers. This precludes them from effectively migrating from the low intensity regions to the high intensity ones, therefore being unable to penetrate and become fixed into these irradiated regions. We need also to consider that, differently from mask-wise exposure, which results in a square-shape intensity profile, the holographic interference pattern generated in our setup has a sinusoidal intensity profile. As a result, if the energy of the pulse increases, the regions closer to the minimum of the interference pattern can also receive energy that exceeds the threshold of crosslinking above which monomer migration is strongly hindered, thus resulting in lower relief structures.

To understand the differences observed in the surface relief height for the three different development temperatures, we need to consider that larger diffusivities of the reactive species can be expected at higher temperatures, favoring the flux of material. In this way, increasing the development temperature from 60 to 80 °C leads to an enhancement of surface relief height, which can be ascribed to the higher diffusional mobility of the monomers. Conversely, further increase in the development temperature to 100 °C leads to lower relief height structures. At too high temperatures, counter diffusion of reactive species from the high-intensity regions to the low ones could be enhanced because of entropic reasons and could convert monomers into polymers already in unwanted, low-intensity locations. Even more, at 100 °C, the termination reactions of radical chain ends could be enhanced, as, not only the diffusion of the monomer is promoted, but also the diffusion of the growing chains. Both effects could contribute to reducing the concentration differences of reactive species between high- and low-intensity regions and thus, reduce surface relief height formation at these elevated temperatures. Also, it is observed (see Figure 2) that higher energy doses are needed to reach the optimum height at this high development temperature of 100 °C. Again, enhanced monomer diffusion at more elevated temperatures can account for this trend as more radicals are needed in the high-intensity regions of the interference pattern to ensure efficient polymerization in this region and thus, a sufficient driving force for the diffusion of monomers to these areas.

### 3.2. Modified Photoembossing Protocol

**Figure 3:**
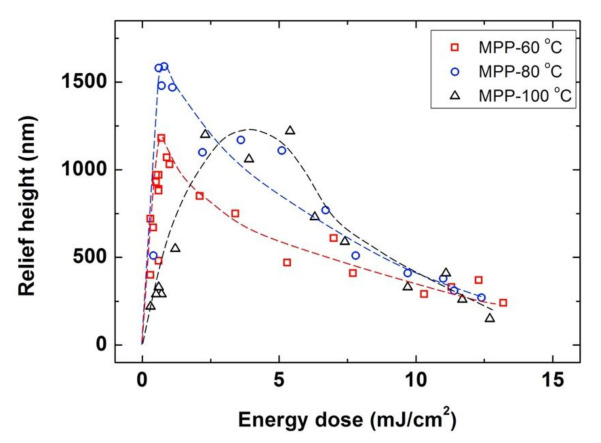
Modified photoembossing protocol (MPP) comprising an exposure step using IL (single pulse exposure and 9 μm period grating) carried out directly at the development temperature. Relief height as a function of energy dose for different development temperatures of 60 °C (**☐**), 80 °C (**◯**) and 100 °C (**△**). Lines are drawn to guide the eye.

After evaluating the formation of surface relief structures using the conventional photoembossing protocol, we explored the preparation of structures by a modified protocol in which the exposure of the sample to the interference pattern was performed directly at the development temperature. Films of the photopolymer were exposed with different doses of energy directly at the development temperature and left 10 min at this same temperature, this being the thermal development step equivalent to that of the conventional photoembossing protocol. After this 10 min, the sample is rapidly cooled down to RT by putting the sample on a metallic block at this temperature. A fixation step of the sample is then carried out by flood-exposing the sample with UV light and later heating at 80 °C for 10 min. Again, it was checked that this fixation step does not introduce changes in the photoembossed structure. Figure 3 gathers the relief heights for samples prepared with different energy exposures carried out at three different development temperatures, namely 60, 80 and 100 °C, using this modified photoembossing protocol. We find qualitatively similar behavior as in the conventional protocol, observing an optimum dose for each development temperature that is higher than 100 °C. Also, the highest relief heights are achieved at 80 °C. Compared to the conventional protocol, photoembossing carrying out exposure directly at the development temperature leads to significantly higher structures having approximately double relief heights. Values up to 1590 nm of height have been reached for an optimum energy as low as 0.8 mJ/cm^2^ when exposing the sample at 80 °C. This is in contrast with the relief height optimum of 750 nm obtained for a higher dose of 3.6 mJ/cm^2^ when using the conventional protocol of exposure at RT and a subsequent thermal development step at 80 °C.

**Figure 4:**
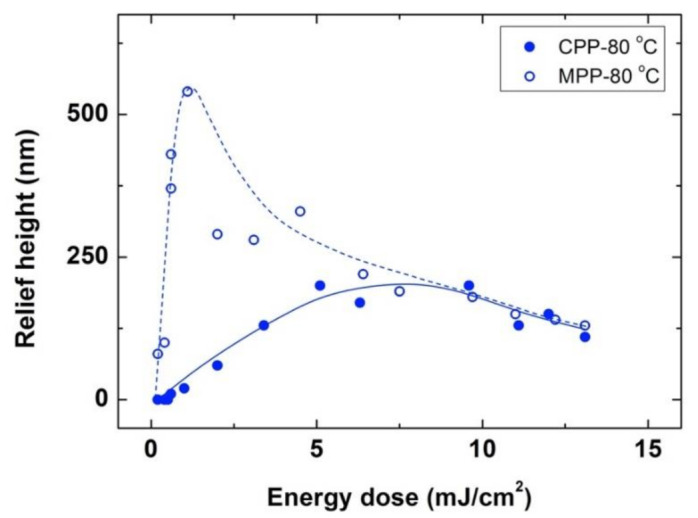
Relief height as a function of energy dose for photoembossed gratings with a 4.5 μm period. The exposure step using IL (single pulse exposure and 4.5 μm period grating) is performed either at RT followed by a development step at 80 °C (**⚫**) or directly at the development temperature (**◯**). In both cases, the samples were kept at this development temperature for 10 min. Lines are drawn to guide the eye.

We have adjusted the angle between the UV interfering beams to generate a smaller periodic sinusoidal pattern of 4.5 μm. Using this optical configuration for the exposure, and similarly to the larger period experiments just shown, photoembossing was carried out by using the conventional and modified photoembossing protocols, as shown in Figure 4. On one hand, exposure at RT and subsequent heating at 80 °C, led to an optimum relief height of approximately 200 nm for doses in the range of 5 to 10 mJ/cm^2^. On the other hand, when the exposure step was directly at the development temperature, at 80 °C, significantly higher surface relief structures, with values up to 550 nm of relief height, were reached for an optimum energy as low as 1.2 mJ/cm^2^. Therefore, qualitatively similar results to those obtained in larger grating period experiments were obtained when exploring these smaller grating periods, attaining significantly higher structures with much lower optimum energies.

To further explore more the processing parameters space, we have studied the influence of the amount of photoinitiator in the formation of surface relief structures using the two studied protocols. For this study, we reduced the amount of photoinitiator in the formulation to 1 wt%, instead of the 5 wt% used in the previously described experiments. The same amount of solvent was used to prepare the photosensitive films by spin coating, obtaining film thicknesses of ≈10 μm, that is, slightly thicker than those obtained with the use of 5 wt% photoinitiator, which were ≈9 μm thick.

**Figure 5:**
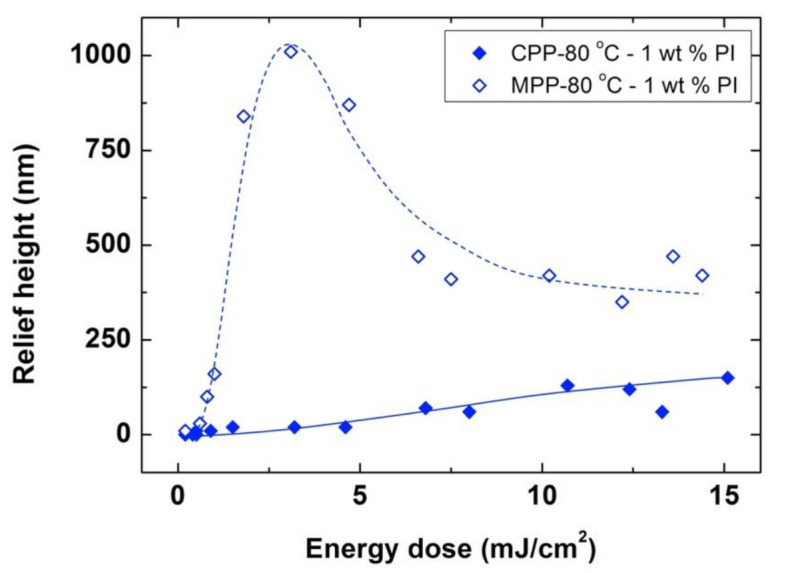
Relief height as a function of energy dose for photoembossed samples bearing 1 wt% photoinitiator. The exposure step using IL (single pulse exposure and 9 μm period grating) is performed either at RT followed by a development step at 80 °C (**◆**) or directly at the development temperature (**◇**). In both cases, the samples were kept at this development temperature for 10 min. Lines are drawn to guide the eye.

Figure 5 presents the surface relief height of structures obtained with different exposure energies for samples of 1 wt% photoinitiator irradiated at RT and heated to 80 °C, as well as those obtained when irradiating directly at 80 °C (heating for 10 min in both cases). While the relief heights obtained for the conventional photoembossing protocol are below 150 nm in all of the explored range of energy doses studied (up to 15 mJ/cm^2^), an optimum height of 1010 nm has been reached for a dose of 3.1 mJ/cm^2^.

Surface relief heights achieved in the samples with 1 wt% photoinitiator are lower than those reached in homologous films with 5 wt% photoinitiator under the same protocol, due to a smaller amount of radicals available after exposure. However, remarkably, the heights obtained in films with 1 wt% photoinitiator, directly exposed to 80 °C are higher than the heights obtained in samples with five times more photoinitiator, if these are exposed to RT and subsequently heated to 80 °C (see Figure 2).

Overall, all the previous results indicate that more efficient use of the photoinitiator is made in the modified photoembossing protocol. Even with substantially lower excitation energies or markedly lower concentration of available photoinitiator, the formation of surface relief structures by polymerization-induced diffusion is much more efficient when excitation is directly carried out at the development temperature. The initiation efficiency of the polymerization process might be at the origin of this difference in the height of the relief structure between protocols. It is known that the free radicals obtained after the irradiation of the photoinitiator have the possibility to recombine, becoming non-effective for the polymerization process [36,37,38]. This possibility of free radical recombination is favored by the collisions suffered by the radicals formed after the photoinduced cleavage process of the photoinitiator with neighboring molecules, a phenomenon known as the cage effect. At RT, with a rigid matrix, the mobility of the photoinduced radicals is hindered, and therefore recombination is favored. This can result in a decrease of the polymerization initiation in the high intensity regions of the interference pattern, resulting in the low incorporation of monomers to the polymeric network in these regions and therefore, in the low surface relief heights of the structure. The probability of recombination is lower at higher temperatures due to the higher molecular mobility existing under these conditions, which provides a greater probability of the photoinduced radicals to escape from the cage, favoring the initiation of polymerization. Thus, the fact that the activation of the photoinitiator is more efficient when exposure is directly carried out at high temperature could be ascribed to the greater possibility of the radicals to escape from the cage, which could account for the high relief modulation reached in the structures prepared under these conditions.

### 3.3. Photoembossed Cell-Guiding Substrates

**Figure 6:**
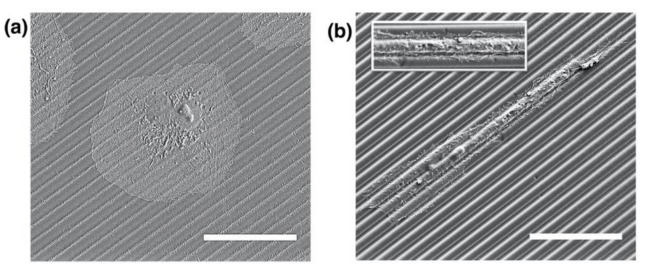
SEM images showing the morphology of kidney epithelial cells (Vero) cultured on 4.5 μm period photoembossed patterned substrates using (**a**) the conventional photoembossing protocol (exposure at RT and development at 80 °C) and (**b**) the modified photoembossing protocol (exposure and development at 80 °C). Inset in (**b**) shows detail of filopodia. Scale bar 30 μm.

Overall it has been demonstrated that higher surface relief structures can be achieved by the modified photoembossing protocol. We have explored the use of these enhanced height surface relief structures for cell culturing. It is well known that the texturing of surfaces strongly affects cell adhesion, proliferation and also, very notably, cell alignment. Here we study the effect of the surface relief height enhancement achieved on cell alignment behavior. For this, we have employed the photopolymer bearing 5 wt% of photoinitiator and selected the optimum conditions spotted for 4.5 μm period photoembossed samples obtained using the traditional and the modified protocols. Kidney epithelial cells (Vero) were cultured on the micropatterned substrates, each of them placed in a different well of a 24-well plate. Cells were incubated for 6 h on the micropatterned substrates. Qualitatively, SEM images (Figure 6) suggest that cells cultured on micropatterned substrates using the conventional photoembossing protocol (exposure at RT and development at 80 °C) show non-preferential alignment. Differently, cells cultured on micropatterned substrates using the modified photoembossing protocol (exposure directly at the development temperature of 80 °C) exhibited clear alignment along the structure lines. Besides alignment, cell morphology seemed also to be different. Cells cultured on substrates obtained using the conventional photoembossing protocol showed a more rounded widespread shape (Figure 6a), while cells cultured on substrates using the modified protocol were more elongated and spindle-like (Figure 6b). Moreover, in this last case, cells extended numerous cell membrane protrusions known as filopodia (see inset of Figure 6b). Filopodia have been reported as sensing organelles of the extracellular environment, playing a key role in cytoskeletal rearrangement and, subsequently, the response to topography through elongation and alignment. These observations are in line with studies carried out in other microstructured systems in which it has been observed that cells present more filopodia when cultured on structured substrates than when they are seeded on flat ones [5,39,40,41,42,43,44,45].

**Figure 7:**
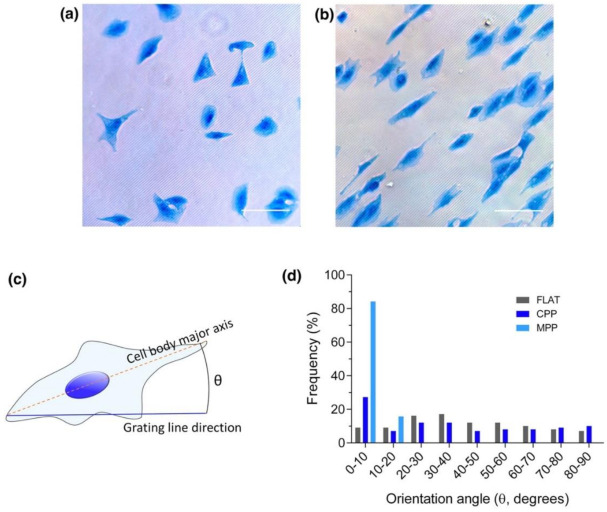
Cell alignment angle analysis on photoembossed substrates. (**a**,**b**) are optical microscopy images of Coomassie blue stained kidney epithelial cells cultured on 4.5 µm period photoembossed patterned substrates using (**a**) the conventional protocol (exposure at RT and development at 80 °C) and (**b**) the modified photoembossing protocol (exposure and development at 80 °C). (**c**) Cell alignment angle was defined as the angle θ between the major axis of the cell body (dashed orange line) and the direction of the substrate grating line (continuous blue line). (**d**) Frequency of cell orientation angle distribution at 10° intervals exhibits cells cultured on flat substrates (FLAT) or patterned ones, using either the conventional (CPP) or the modified photoembossing protocol (MPP). Scale bar 100 μm.

For the observation of the cells under the optical microscope, as detailed in the experimental section, cells were additionally stained for non-specific proteins with Coomassie blue to enhance the contrast and better visualize cell morphology and elongation state. Figure 7a,b shows optical microscopy images of stained cells cultured on 4.5 μm period structures using conventional (Figure 7a) and modified (Figure 7b) photoembossing protocols. Cell alignment was defined as the angle, θ, between the major axis of the cell body and the direction of the photoembossed lines (Figure 7c). Cell alignment angles with respect to the grating lines were determined for the observed cells and reduced to a single quadrant between 0° and 90°. The number of cells found for each ten-degree interval in this range is represented in Figure 7d. As expected, no significant preferential alignment is identified in a flat non-structured reference sample. A slight alignment is, however, found for cells cultured on 4.5 μm photoembossed substrates obtained using the conventional protocol (surface relief height of 200 nm) with 27% of the cells within the 0° to 10° interval. This percentage is approximately double when compared with those found at the rest of the intervals of 10° (see Figure 7d). Remarkably, when cells are cultured on micropatterned substrates processed under the modified photoembossing protocol with exposure and development carried out at 80 °C (surface relief height of 550 nm), cells preferentially align along the direction of the grating lines. A percentage (84%) of the cells are found between 0° and 10°.

Trying to reduce the obtained cells’ orientation distribution to one single scalar, we define an order parameter *S* (Equation (1)), as is done when dealing with ensembles of anisotropic objects lying in a plane and aligned along a preferential direction [46].

(1) 〈S=cos2θ〉

For perfectly aligned cells, we would have *S* = 1, while, for randomly oriented ones, *S* = 0. With this definition, an order parameter S of 0.28 is calculated for cells cultured on micropatterned substrates processed under the conventional photoembossing protocol. The control flat sample led to an order parameter of 0.09, closer to the perfectly disordered distribution (*S* = 0). A significantly elevated *S* value of 0.94 is reached for cells cultured on the micropatterned structures obtained though the modified photoembossing protocol, further demonstrating the strong effect of the surface relief enhancement obtained on cell alignment.

**Figure 8:**
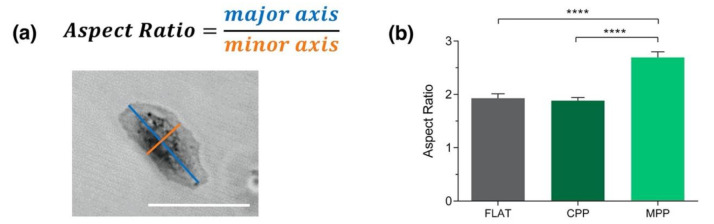
(**a**) Aspect ratio is defined as the ratio of the cellular major and minor axes. Scale bar 30 μm. (**b**) Cell morphology analysis on flat substrates (FLAT) or patterned ones, using either the conventional (CPP) or the modified photoembossing protocol (MPP). Unpaired Student’s *t*-test **** *p* < 0.0001.

Besides alignment, we have also analyzed cell morphology in these experiments. The aspect ratio was defined as the major cell axis divided by the minor one (Figure 8a). Cells cultured on 550 nm height substrates (MPP) showed an increased aspect ratio compared to 200 nm height substrates (CPP) and flat control (Figure 8b), showing a more elongated spindle-like geometry as aforementioned and shown in Figure 6 and Figure 7. Interestingly, the higher the aspect ratio, the greater the cell alignment, indicating that substrate height plays an important role in cell morphology and migration.

The way cells react to different topographies in terms of orientation, shape, movement and functionality is a topic widely studied in the literature. As already mentioned, it has been extensively demonstrated that increasing the depth of the grating grooves enhances cell orientation along structure lines [47], as corroborated by our results. However, little is known about the specific molecular events that trigger this behavior. The experiments carried out in this paper have been focused on testing, for the first time, how the cells react to this material when structured using nano-second laser interference photoembossing and, once we have revealed the good cellular orientation in the range of periods and depths achieved, further experiments could be designed to elucidate cell behavior at the molecular level. For example, there is some evidence that correlates the polarization of cells over microstructures with the orientation of the extracellular matrix formation. In this line, it has been described that, in the case of osteoblasts, while the cells were oriented along the nanogrooves, the collagen matrix deposition was perpendicular to the grooves, an effect that was facilitated by focal adhesion formation and maturation [48,49]. To better understand how cells sense differently the two microstructures used in this manuscript (low vs. high relief height, 200 vs. 550 nm), leading to such different cell orientations and cell shapes, further experiments need to be carried out. The focal adhesions formation in both cases could be analyzed by immunofluorescence staining or, trying to achieve better resolution, with immunogold labeling [50]. Comparing the number, length and the orientation of the focal adhesions would help us to better understand cell behavior at a molecular level when cells adhere to the tested microstructures [51]. Despite this and other more specific assays being out of the scope of this paper, we present them to highlight the interest of the proposed photoembossing protocol that could give access to a variety of polymeric relief gratings with tunable grating topographies of interest for these fundamental cell alignment studies.

## 4. Conclusions

In this paper, it was shown that photoembossed structures obtained through a pulsed holographic exposure directly carried out at the surface relief development temperature present enhanced relief with respect to those prepared by performing a conventional RT exposure followed by thermal development. Optimum relief heights reached using the high-temperature exposure-modified photoembossing protocol are more than double than those obtained carrying out exposure at RT. Advantageously, in the modified protocol the energies required to reach the optimum dose are also much lower (four times lower); furthermore, the amount of photoinitiator in the formulation can be substantially reduced while keeping high relief heights. This could be advantageous in biomedical applications in which this formulation additive can potentially lead to toxicity effects. Overall, better efficiency of the surface relief generation process is demonstrated when exposure is directly carried out at high temperatures that could be ascribed to better efficiency in the polymerization initiation reaction. Enhanced cell alignment is found for substrates with relief-height-optimized polymeric gratings generated with light exposure at the development temperature when compared to those obtained with RT exposure and the subsequent thermal development step, highlighting the relevance of the newly introduced process. The higher height achieved enhances the cell alignment following the patterns then polarizing better the cell elongation. Despite it is well-known that increasing the relief height of the polymeric grating enhances the cell orientation along the lines, as seen in this work, we have here demonstrated how photoembossing, using the proposed modified protocol, provides easy access to polymeric relief gratings with tunable periods and relief heights in the range of interest for this type of cell orientation study. In this same direction, we could also benefit from the flexibility to change material chemistry using this structuring technique, making nano-second laser interference photoembossing an attractive platform to generate *a la carte* microstructured substrates for fundamental biological studies.

## Data Availability

Data supporting reported results can be requested to the corresponding author.
